# The Disastrous Effects of Salt Dust Deposition on Cotton Leaf Photosynthesis and the Cell Physiological Properties in the Ebinur Basin in Northwest China

**DOI:** 10.1371/journal.pone.0124546

**Published:** 2015-05-13

**Authors:** Jilili Abuduwaili, Zhang Zhaoyong, Jiang Feng qing, Liu Dong wei

**Affiliations:** 1 State Key Laboratory of Desert and Oasis Ecology, Xinjiang Institute of Ecology and Geography, Chinese Academy of Sciences, Urumqi, China; 2 University of the Chinese Academy of Sciences, Beijing, China; 3 College of Environment and Resources, Inner Mongolia University, Hohhot, China; Louisiana State University Agricultural Center, UNITED STATES

## Abstract

Salt dust in rump lake areas in arid regions has long been considered an extreme stressor for both native plants and crops. In recent years, research on the harmful effects of salt dust on native plants has been published by many scholars, but the effect on crops has been little studied. In this work, in order to determine the impact of salt dust storms on cotton, we simulated salt dust exposure of cotton leaves in Ebinur Basin in Northwest China, and measured the particle sizes and salt ions in the dust, and the photosynthesis, the structure and the cell physiological properties of the cotton leaves. (1) Analysis found that the salt ions and particle sizes in the salt dust used in the experiments were consistent with the natural salt dust and modeled the salt dust deposition on cotton leaves in this region. (2) The main salt cations on the surface and inside the cotton leaves were Na^+^, Ca^2+^, Cl^-^ and SO_4_^2-^, while the amounts of CO_3_^-^ and HCO_3_^-^ were low. From the analysis, we can order the quantity of the salt cations and anions ions present on the surface and inside the cotton leaves as Na^+^>Ca^2+^>Mg^2+^>K^+^ and Cl^-^>SO_4_^2-^>HCO_3_^-^>CO_3_^-^, respectively. Furthermore, the five salt dust treatment groups in terms of the total salt ions on both the surface and inside the cotton leaves were A(500g.m^-2^)>B(400g.m^-2^)>C(300g.m^-2^)>D(200g.m^-2^)>E(100g.m^-2^)>F(0g.m^-2^). (3)The salt dust that landed on the surface of the cotton leaves can significantly influence the photosynthetic traits of *P_n_, PE, C_i_, T_i_, G_s_, T_r_, WUE, L_s_, φ, A_max_, k* and *R_ady_* of the cotton leaves. (4)Salt dust can significantly damage the physiological functions of the cotton leaves, resulting in a decrease in leaf chlorophyll and carotenoid content, and increasing cytoplasmic membrane permeability and malondialdehyde (MDA) content by increasing the soluble sugar and proline to adjust for the loss of the cell cytosol. This increases the activity of antioxidant enzymes to eliminate harmful materials, such as the intracellular reactive oxygen and MDA, thus reducing the damage caused by the salt dust and maintaining normal physiological functioning. Overall, this work found that the salt dust deposition was a problem for the crop and the salt dust could significantly influence the physiological and biochemical processes of the cotton leaves. This will eventually damage the leaves and reduce the cotton production, leading to agricultural economic loss. Therefore, attention should be paid to salt dust storms in the Ebinur Basin and efficient measures should be undertaken to protect the environment.

## Introduction

**Fig 1:**
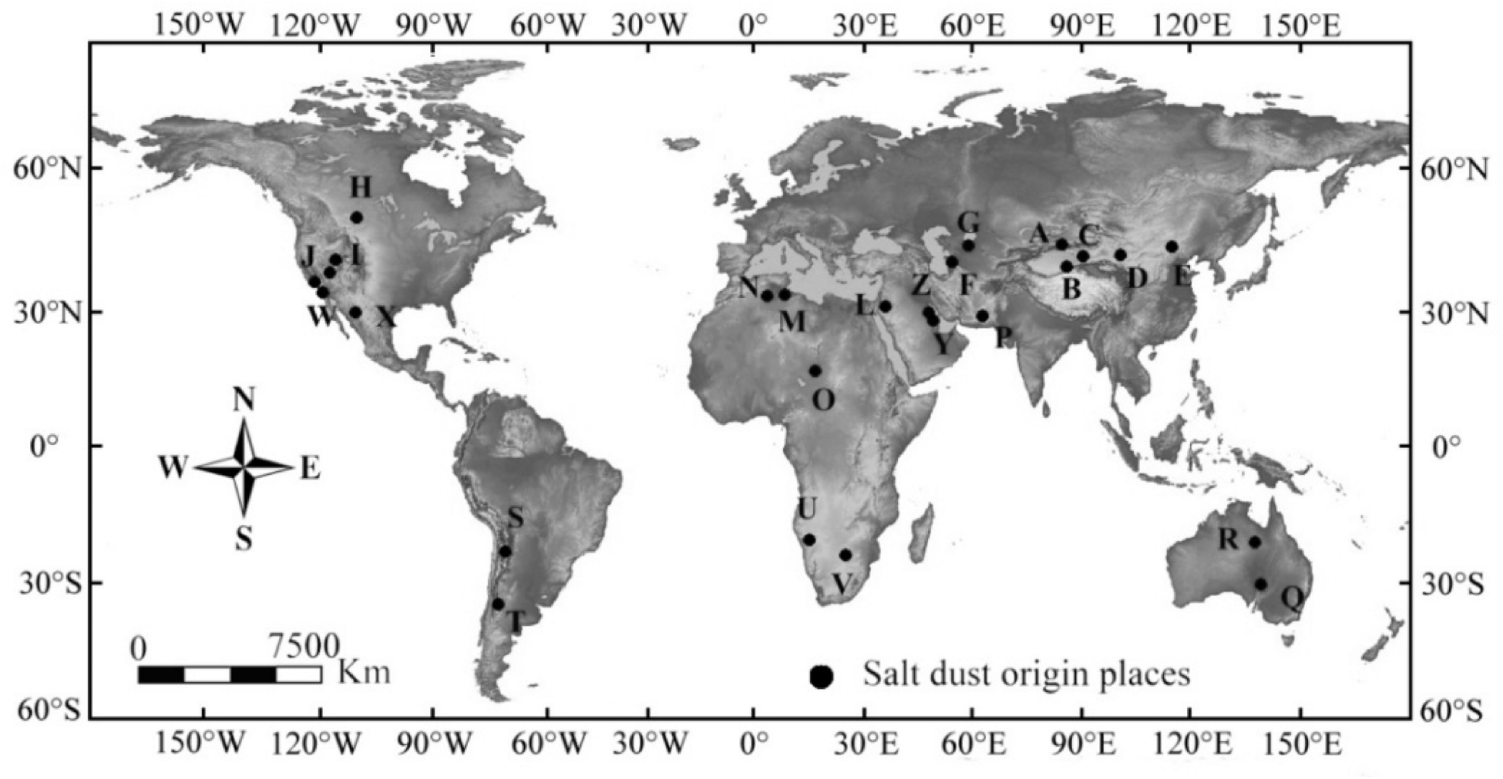
Distribution of saline lakes, playas and similar landforms in arid and semiarid areas susceptible to salt dust storms.

Salt dust storms are extreme weather phenomena that primarily originate from wind erosion of dried up salt ion rich lake sediments (Fig 1) [1–6]. These salt dust storms differ from the typical sand storms in that they contain a high density of very small particles of sulfate, chloride, pesticide dust and harmful heavy metals, including Pb, Cu, Cr, Hg and Zn [7–9]. The frequent occurrence of salt dust storms within the Ebinur Basin has led to a large amount of scattering of saline dust to the surrounding areas, which can cause desertification of lakesides and plains, damage vegetation and catalyze the formation of new desert [10]. Salt dust is also a serious air pollutant that is very harmful to human health. Furthermore, the frequent occurrence of salt dust storms in the Ebinur Basin has negatively impacted railway traffic, even necessitating suspension of railway service [1,9–11]. In agriculture, the salt dust pollution can affect soil quality, available soil nutrients and minerals, organic matter, pH and amount of clay [12–14]. Salt dust storms can also significantly influence plant height, root length and seed germination rate, and cause changes in root, stem and leaf morphology, such as a decrease in fresh weight [1,10,15,16].

When salt dust lands on plant leaves, the leaf surface becomes covered, which captures the plant’s moisture and blocks the stomata, respiration and photosynthesis that normally occurs. This salt dust covering shades the leaves from radiation from the sun, affects photosynthesis and leaf reflectance, increases the leaf temperature, affects the surface humidity, gas exchange and assimilation of the blades, and influences pollination [9,17,18]. This can result in degeneration in the plant’s ability to function, decreasing the biomass and yield and, thus, causing serious agricultural economic loss [4,19].

Previous studies have tended to focus on plants in high and low temperatures, drought, stress caused by cement and fly ash dust, and crops including wheat, cotton, corn and vegetables. The studied parameters included amount of leaf chlorophyll, changes in leaf cell structure, blade internal physiological indexes of cytoplasm membrane permeability, MDA content, active oxygen changes, and substances involved in osmotic regulation, such as proline, betaine and soluble sugar, and antioxidant enzymes, such as superoxide dismutase (SOD), peroxidase (POD) and catalase (CAT). However, there have been no reports concerning the impact of salt dust on cotton crops, including on the salt ion distribution on the cotton leaves, and the internal and structural changes to the cotton leaf under salt dust deposition.

Xinjiang is China's major cotton producing region, and is worldwide famous for its high quality cotton production [20]. The Ebinur Basin is located in the south of the famous Alataw Pass in Northwest China. In this region, the wind speed can be greater than 8 m.s^-1^ up to 164 days of the year with a maximum instantaneous wind speed of 55 ms^-1^ throughout the year [1,21]. Since the 1950s, the development of industrial and agricultural production in the Ebinur Basin has resulted in large quantities of water resources being used for irrigation without a corresponding increase in the amount of precipitation. This has led to a dramatic decrease in the amount of water in Ebinur from 1200 km^2^ to 500 km^2^, lowering the water level of the lake and drying up areas. This dried bare lake bottom has caused serious ecological problems in this basin [2,3,22,23]. The loose sediment at the bottom of the dry lake is rich in salt ions that are susceptible to wind erosion at wind speeds as low as 4.3–6.4 m.s^- 1^. The strong winds from Alataw Pass can easily blow about this loose sediment and spread the salt dust (particle size often 2–63 μm as measured by Liu et al. [1]. This salt dust can cause a disastrous salt storm and can be transported up to thousands of kilometers away. The Ebinur Basin is a famous salt dust source in Northwest China and central Asia [6,24].

Since the 1970s, the frequent occurrence of salt dust storms in the Ebinur Basin has seriously harmed the surrounding environment. Amounts this high of salt dust have damaged the vegetation, reduced agricultural production, desertified grassland and the surrounding lake plain, and accelerated the formation of new desert. However, current studies still fail to assess the impact of salt dust storms on agriculture, including on the main crop of the Ebinur Basin and arid regions, cotton. Simulation experiments were used in this work to study the influence of salt dust on the salt content of the cotton leaves, particle size distribution, photosynthesis, leaf blade cell structure, amounts of chlorophyll, proline, soluble sugar and MDA, and antioxidant enzyme activity. From this, we can provide a scientific basis for studying the salt dust storms in this region and other similar regions around the world, and then take proper measures to protect the environment.

### Experimental Setup and Data Acquisition

**Fig 2:**
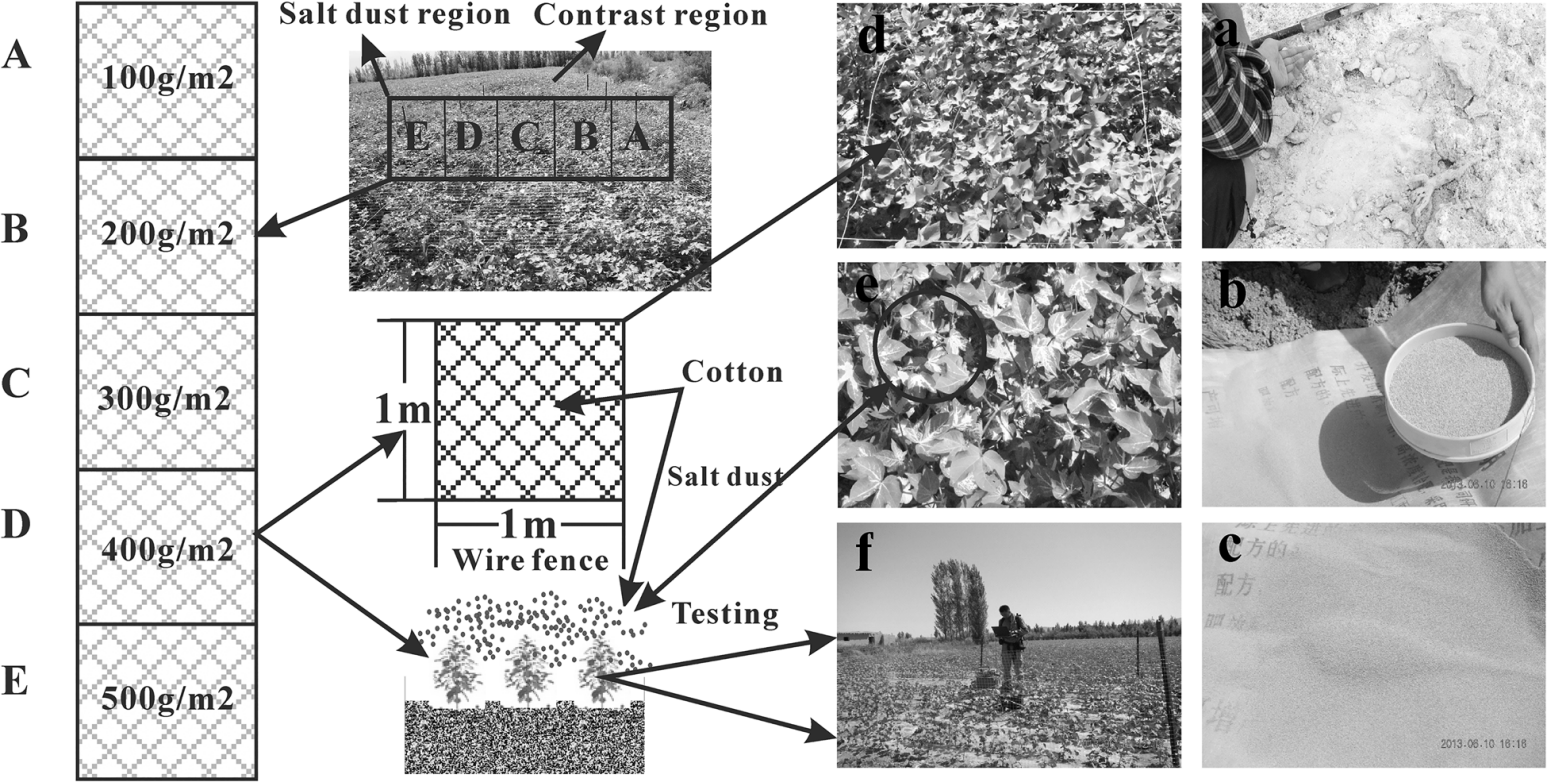
Setup of simulation experiments.

The sample collection and test were all permitted in our country and no specific permissions were required for these locations/activities, we confirm that the field studies did not involve endangered or protected species. The salt dust for the experiments was obtained from the Alataw Pass in Xinjiang in Northwest China as follows. First, loose saline soil was broken up and ground up, then pushed through a fine screen (0.02 mm). Then, under windless and sunny conditions on June 6^th^, 2013, we ran an artificial simulation of salt dust deposition consisting of five salt dust spray gradients of 0 g.m^-2^, 100 g.m^-2^, 200 g.m^-2^, 300 g.m^-2^, 400 g.m^-2^ and 500 g.m^-2^ in the cotton fields in the southwest of Jinghe county in the Ebinur Basin, China (Fig 2), and four-replication groups of each salt dust simulated level were settled.

After allowing the experiment to run for 15 days (June 21^th^, 2013), the salt dust that had landed on the cotton leaves was collected. From this, the particle sizes, composition of the salt ions on the surface and inside the leaves, and photosynthesis of the leaves (using parameters of the net photosynthetic rate (*P*_n_), light use efficiency (*PE*), intercellular CO_2_ concentration (*C*_i_), leaf temperature (*T*_i_), stomatal conductance (*G*_s_), transpiration rate (*T*_r_), water use efficiency (*WUE*), limiting value of stomata (*L*_s_) and the light response) were all measured and each value was chosen as the average of four-replication groups of each salt dust simulated level.

The *WUE* was calculated as the ratio of the net photosynthetic rate (*P*_n_) and the *T*_r_. The limiting value of the stomata (*L*_s_) was calculated as *C*_a_-*C*_i_/*C*_a_×100%, in which *C*_a_ is the concentration of atmospheric CO_2_ and *C*_i_ is the intercellular CO_2_ concentration. From there, we calculated the light response parameters of the cotton leaves each day.

### Determination of salt dust particle size distribution on the cotton leaves

To determine the particle size of the salt dust on the cotton leaves, the following steps were taken. First, 30% hydrogen peroxide was added at 72°C to remove any organic matter from the salt dust, which had been collected from the surface cotton leaves in the monitoring area using a fine wool brush. We added hydrochloric acid to remove the carbonate, then ultrapure water to dilute the solution and let it stand for 30 min to remove the supernatant acid. Next, quieted it over and over again until the pH reached 6.5–7.0. Finally, we added sodium hexametaphosphate after 30 min of ultrasound dispose, we used a Mastersizer 2000 (Melvin laser particle size analyzer, USA) with a range of 0.02–2.000 μm to measure the particle sizes of the salt dust.

### Measurement of the salt ions on the surface and inside cotton leaves

In this work, the salt ions on the surface of the cotton blade were collected in water used to rinse the cotton leaves. First, we collected the cotton leaves and put them into deionized water for 2–4 min to rinse off the surface particles into fluid. Then, the Na^+^ and K^+^ contents in the rinsing water were tested by FD640 type flame photometer, Ca^2+^ and Mg^2+^ were tested with a general TAS990 type flame atomic absorption spectrophotometer, Cl^-^ was tested using nitric acid silver titration, CO_3_^2-^ and HCO^-^ were tested using the double indicator method and the SO_4_^2-^ was tested using EDTA titration [25].

The salt ions inside the cotton blade were collected by boiling the blade in liquid. After the cotton leaves were rinsed as described above, removing any salt ions on the surface of the leaves, we boiled them for 30 min to extract the salt ions inside the cotton leaves. Then, the Na^+^ and K^+^ contents in the rinsing water were tested by FD640 type flame photometer, Ca^2+^ and Mg^2+^ were tested with a general TAS990 type flame atomic absorption spectrophotometer, Cl^-^ was tested using nitric acid silver titration, CO_3_^2-^ and HCO^-^ were tested using the double indicator method and the SO_4_^2-^ was tested using EDTA titration [25].

### Measurement of photosynthesis by the cotton leaves

The photosynthesis of the cotton leaves was measured on a sunny day from 10:00–20:00. The test equipment was a Li-6400 portable photosynthesis measuring system (Li-Cor, Lincon, NE, USA). The tested indicators of photosynthesis were the *P*_n_, *PE*, *C*_i_, *T*_l_, *G*_s_, *L*_s_, *T*_r_ and *WUE*. During the test, each of the above parameters was tested three times for each sediment group per hour from 10:00 to 20:00, and each value of them was chosen as the average of the four-replication groups of each salt dust simulated level (from 0 g.m^-2^ to 500 g.m^-2^). We then calculated the average value of these three values to obtain the final test value. Next, the *L*_s_ and *WUE* of the cotton leaves were calculated as (*C*_a_-*C*_i_)/*C*_a_×100% and *P*_n_/*T*_r,_ respectively.

### Generation of light response curves of the cotton leaves

Light response curves were calculated using the nonrectangular hyperbola model with relevant parameters and a derivation of equation fitting of *P*_n_ and *PAR*. First, we used the model to calculate the parameters of the curved angle (*K*), the apparent quantum efficiency (*θ*), the maximum net photosynthetic rate (*A*_*max*_) and the respiratory rate (*R*_*ady*_). Then, we calculated the light linear equation of effective photosynthetic radiation under 200 μmol.m^-2^.s^-1^ and the intersection of these two straight lines.

### Leaf blade surface morphology

Cotton leaves where salt dust had landed and where no salt dust had landed were collected. These leaves were then dried and processed according to standard scanning electron microscopy (SEM) biological sample preparation protocols. An IB-5 Ion sputtering apparatus plating was used to pin gold film, and a scanning electron microscope with type LECM430VP was used to observe and photograph the cotton samples.

### Measurement of chlorophyll content

The acetone grinding extraction method was used to measure the chlorophyll content of the cotton leaves [27]. Briefly, 80% acetone was used to extract fluid for testing, a type 756MC ultraviolet-visible spectrophotometer was used to measure the absorbance (D) at wavelengths of 663, 646 and 470 nm, and then the total chlorophyll content was calculated. Each value of them was chosen as the average of the four-replication groups of each salt dust simulated level.

### Measurement of the carotenoid content in the cotton leaves

First, 2 g of cotton leaves were ground after freezing with liquid nitrogen. Next, 8 mL of acetone were added to the extraction and incubated at 4°C in the dark. After 24 h, the mixture was centrifuged at 10,000 g for 5 min and the supernatant was transferred to a new 10 mL centrifuge tube, then 7 mL of acetone were added to the residue. The extraction was repeated 3 times and all the supernatants were added together. Acetone was added to the supernatants to make a total volume of 25 mL. Prior to testing, the solution was passed through a 0.22 mm organic filter head. Take extracting solution of 10 mL, the total carotenoid content in the solution was then measured using a UV755B ultraviolet-visible spectrophotometer at a wavelength of 450 nm and calculated based on these measurements [28].

### Measurement of the soluble sugar in the cotton leaves

The anthrone colorimetry method was used to test the soluble sugar content of the cotton leaves [29]. First, 1 mL extraction liquid was prepared, mixed with 5 mL anthrone reagent, put in a boiling water bath for ten min, and then cooled. A 756MC ultraviolet-visible spectrophotometer was used to measure the absorbance (D) at a wavelength of 625 nm. The absorbances were compared to the absorbances of different concentrations of standard solutions and then the soluble sugar concentration curves were drawn.

### Measurement of the proline content of the cotton leaves

The acidic indene three ketone chromogenic method was used to measure the proline content of the cotton leaves under different salt dust deposition conditions. First, l mL fluid was prepared for the test, and then 5 mL glacial acetic acid was added, followed by 5 mL indene three ketone color liquid. This solution was incubated at a constant 80°C in a water bath for 40 min, then cooled to room temperature and shaken well with distilled water constant volume. Finally, an ultraviolet-visible spectrophotometer was used to measure the absorbance (D) at a wavelength of 520 nm. These obtained absorbances were compared to the absorbances at different concentrations of a standard solution, and a proline concentration curve was drawn and used to calculate the proline content in the fluid [29].

### Measurement of the MDA content

The MDA content of the cotton leaves was measured using the glucosinolates barbituric acid method under different salt dust deposition conditions. First, extraction was performed by grinding cotton leaves in an ice bath. 2 mL of 0.67% glucosinolates barbituric acid were added, the solution was mixed and boiled in a water bath for 30 min, and then centrifuged at 3,000 rpm for 10 min after cooling. Finally, an ultraviolet-visible spectrophotometer was used to measure the absorbance (D) at wavelengths of 450, 532 and 600 nm, and these values were used to calculate the MDA content [29].

### Measurement of antioxidant enzymes in the cotton leaves

#### Superoxide dismutase (SOD)

The nitroblue tetrazolium (NBT) method was used to measure the SOD content of the cotton leaves under different salt dust deposition conditions. First, the leaf samples were ground in an ice bath, then prepared for testing by mixing with the reaction liquid and letting the color develop under a 4,000 Lx fluorescent lamp. After 20 min, color comparisons were made and an ultraviolet-visible spectrophotometer was used to measure the absorbance (D) at a wavelength of 560 nm. These values were converted into the SOD content [30].

#### Peroxidase (POD)

The guaiacol method was used to measure the POD content of the cotton leaves. First, the leaf samples were ground in an ice bath. Then, the ground leaves were mixed with a reaction liquid, incubated for 3 min in a 34°C water bath, and an ultraviolet-visible spectrophotometer was used to measure the absorbance (D) at a wavelength of 470 nm at consecutive time points over 4 min. By comparing these values to the standard curve, the POD content was determined [27].

#### Catalase (CAT)

The guaiacol method was used to measure CAT content of the cotton leaves. First, the cotton leaves were ground in an ice bath, and then mixed with a reaction liquid and 300 μL deionized water. Finally, an ultraviolet-visible spectrophotometer was used to measure the absorbance (D) at a wavelength of 240 nm, making consecutive measurements over 4 min. By comparing the values to the standard curve, the CAT content was obtained [27].

### Statistics

To determine whether there is significant influence of five salt dust deposition groups and the control group for the distribution of salt ions and measure the photosynthesis in the cotton leaves, the One-way analysis of variance (ANOVA) (P<0.05) was used. Then if the ANOVA indicated significant treatment effects, the LSD test was used to test for the differences among these treatments.

In this research, data processing was performed using the software SPSS 19.0 and Matlab 7.0, while all work with images was done with CorelDraw 12.0.

## Results and Analysis

### Particle size distribution and salt ion content of the salt dust on the cotton leaves

#### Particle size distribution of the salt dust on and the surface morphology of the cotton leaves

**Fig 3:**
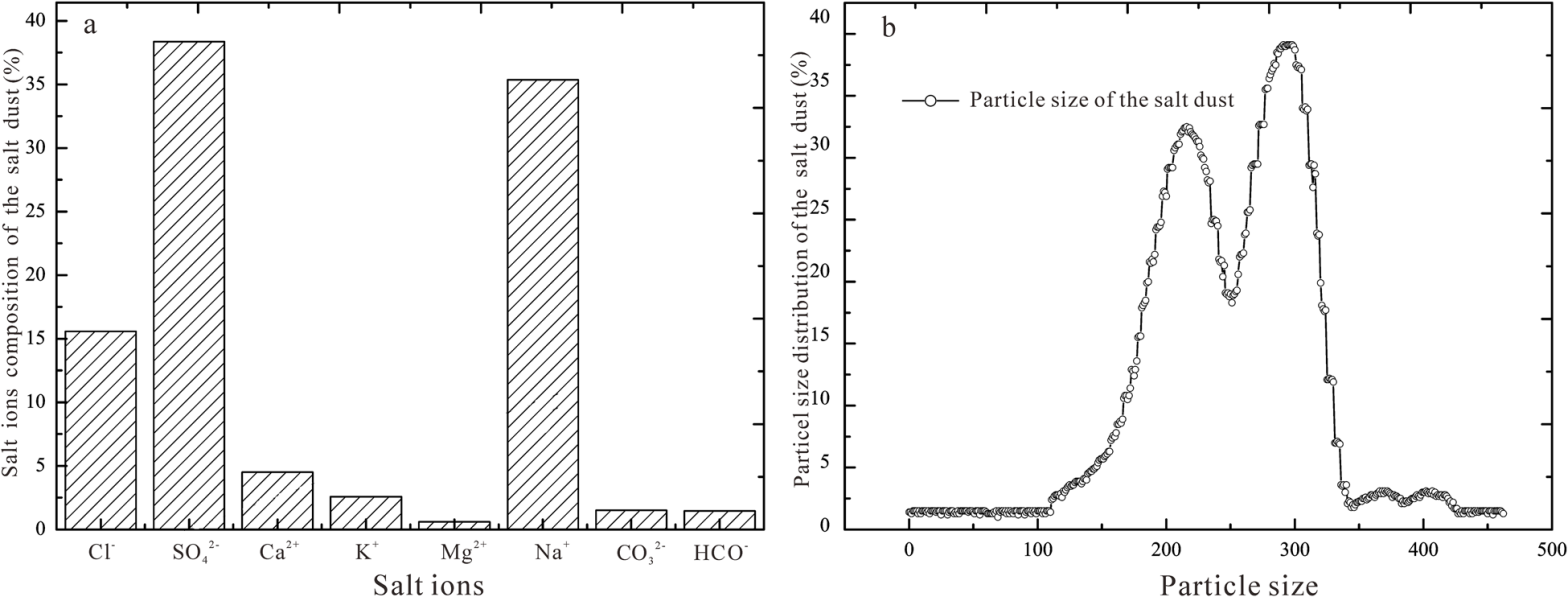
Salt ion fractions in (a) and particle size distributions (b) of the salt dust (μm).

**Fig 4:**
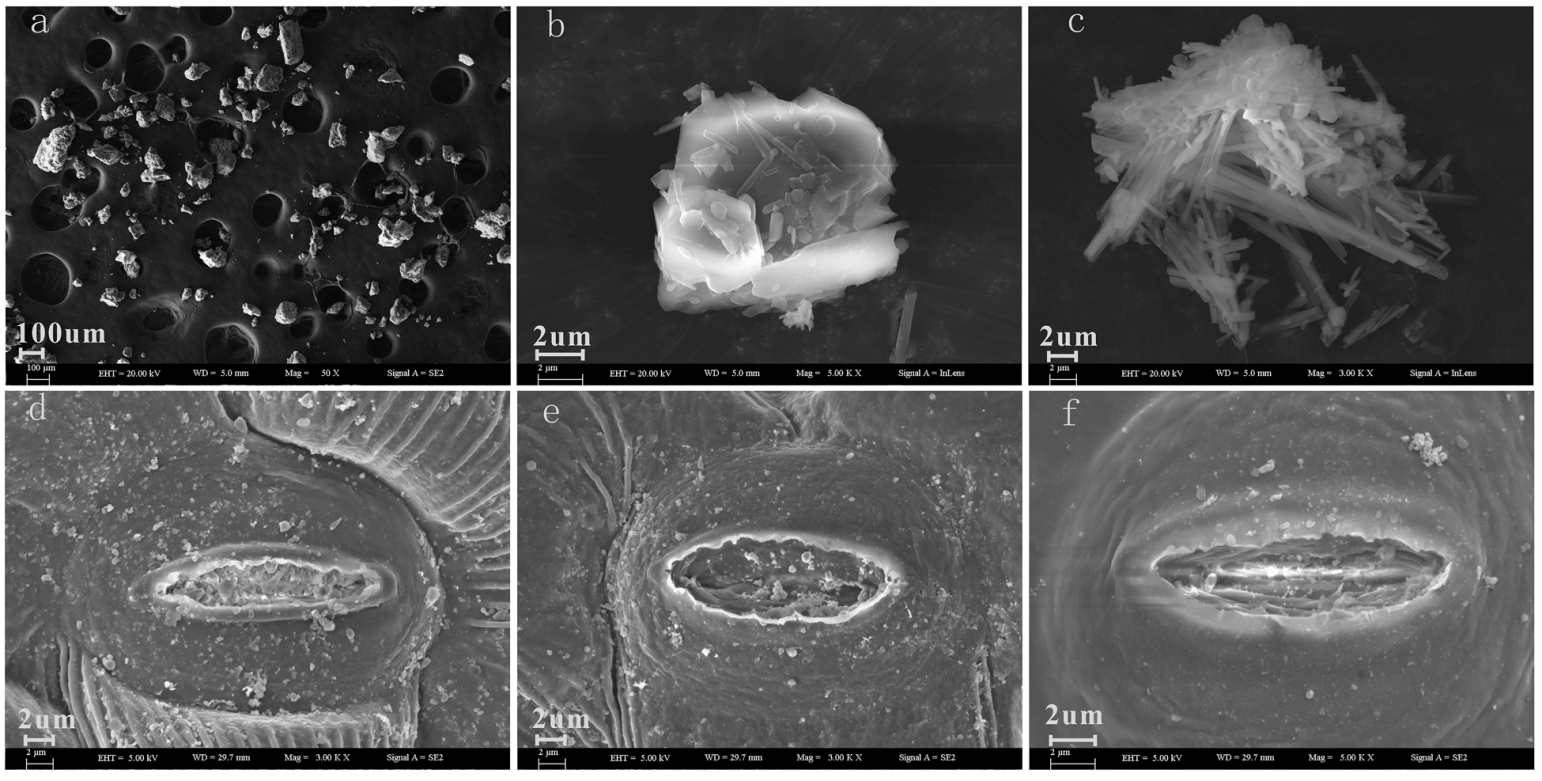
The surface morphology of the cotton leaves.

(1) Overall, the salt dust for this experiment totaled 465 g.k^-1^g with Na^+^ and Ca^2+^ accounting for 39.9% of the salt ions and Mg^2+^ and K^+^ only accounting for 3.18% (Fig 3a). The water-soluble anions consisted mainly of Cl^-^ and SO_4_^2-^, which were 53.94%, while CO_3_^2-^ and HCO_3_^-^ were only 2.97%. Measuring the particle size of the salt dust that landed on the surface cotton leaves (Fig 3b) showed that for the salt dust that had a total mass of 389 g.kg^-1^, the main salt cations were Na^+^ and Ca^2+^ (34.9%), the water-soluble anions were mainly Cl^-^ and SO_4_^2-^,(45.94%), and the salt ions of Mg^2+^ and K^+^ were 14.13%. The particles sized 1.02–58 microns, the silty sand fraction, made up 69.48%, less than 4 microns made up 12.5%, and more than 58 microns made up 27.02%. Overall, these data indicate that the salt dusts used for experiments reflected the natural salt dust found on the cotton plants in the region of interest.

(2) By using the Field Emission Scanning Electron Microscope (FESEM) (ZEISS SUPRA55 VP, Germany), we observed that the stomata of the cotton leaves were labiate with a long axis and a short axis (Fig 4d, 4e and 4f). The particle size range of the salt dust that landed on the cotton leaves was predominantly 0–30 μm (Fig 4a, 4b and 4c) with a width of 1–6 μm. SEM also showed that the length of the stomata of the cotton leaves was mainly 6–12 μm and the width was 1.5–3.5 μm, demonstrating that the salt dust within this size range can easily fall into the stomata. These data also show that the shape of the dust widely varies, and when salt dust within this range lands on the surface of the cotton leaves and covers the blade, it can form a covered area that influences the physiological functions of the cotton leaves, including photosynthesis and cellular respiration, and elements such as salt ions may increase the salt content in the cotton leaves. Thus the internal physiological functions of the cotton leaves can be influenced as well, leading to damage to the cotton leaves.

### Distribution of the salt cations on the cotton leaves

**Fig 5:**
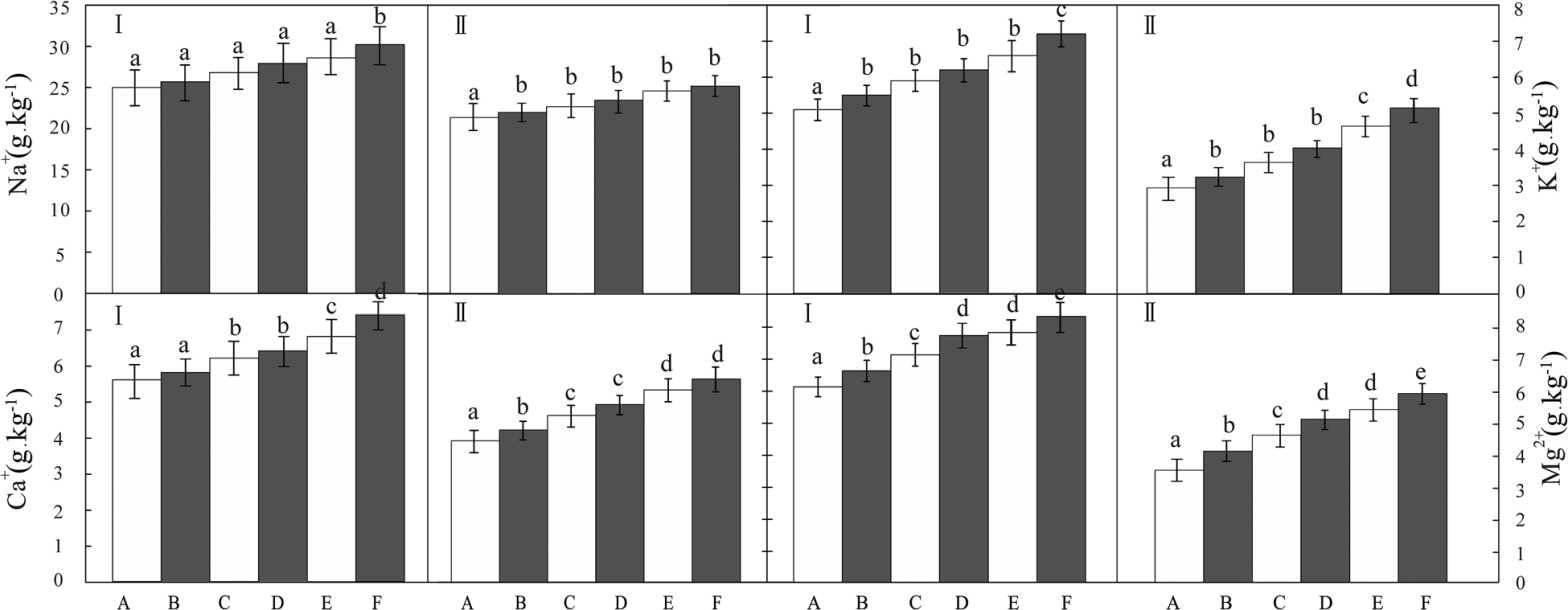
Distribution of the salt cations on the cotton leaves.

From the analysis (Fig 5), we determined the order of the salt cations on the surface of the cotton leaves of the control and five salt dust treatment groups as: Na^+^>Ca^2+^>Mg^2+^>K^+^ and the order of salt ions inside the cotton leaves as: Na^+^>Ca^2+^>Mg^2+^>K^+^. Furthermore, the analysis showed the total salt content on the surface of and inside the cotton leaves corresponded to the treatment groups, which was ordered as: 500 g.m^-2^>400 g.m^-2^>300 g.m^-2^>200 g.m^-2^>100 g.m^-2^.

ANOVA showed that the salt dust deposition had significant influence on the salt cation contents of the surface cotton leaves (*P*<0.05), and the differences among these groups are significant. Then the further LSD test showed that by comparing the treatment groups to the control, it was found the Na^+^ content on the surface of the cotton leaves trended an increase in all the five treatment groups, comparing with the control group. There were significant differences between groups A, B, C, D, or E and F (*P*<0.05), but there were no significant differences between groups A, B, C, D, and E. Compared with the control, the K^+^ content on the surface of the cotton leaves also increased in the five salt dust treatment groups with significant differences found between groups A and (B, C, D, and E), and F (*P*<0.05), but no significant differences were found between groups B, C, D, and E) (*P*>0.05). For the Na^+^ content inside the cotton leaves, compared with the control, there were significant differences between groups of A and B, C, D, E, or F) (*P*<0.05), but there were no significant differences between the groups of B, C, D, E, and F). For the K^+^ content inside the cotton leaves, compared with the control, there were significant differences between groups A and (B, C, and D), and E, and F (*P*<0.05), but there were no significant differences between groups B, C, and D.

As compared to the control, the Ca^2+^ content on the surface of the cotton leaves increased in the five salt dust treatment groups. Compared with the control, there were significant differences between groups (A, B) and (C, D), and E, and F (*P*<0.05), but there were no significant differences between groups (A, B) and (C, D), respectively, (*P*>0.05). Inside the cotton leaves, there were significant differences between groups A, and B, and (C, D), and (E, F), as compared to the control (*P*<0.05), but there have no significant differences between the groups of (C, D) and (E, F), respectively. As compared to the control, the Mg^2+^ content on the surface of the cotton leaves increased in the five salt dust treatment groups. There were significant differences between groups A and B, and C, and (D, E) and F (*P*<0.05), but there were no significant differences between the salt dust groups D and E. A similar trend was observed inside the cotton leaves. There were significant differences between groups A and B, and C, and (D, E) and F as compared to the control (*P*<0.05), but no significant differences were observed between the salt dust groups D and E.

I is on the surface of the cotton leaves; IIis inside of the cotton leaves

The control (A), and groups treated with 100g.m^-2^ (B), 200g.m^-2^ (C), 300g.m^-2^ (D), 400 g.m^-2^ (E), and 500 g.m^-2^ (F). Samples within one index with different lowercase letters a, b, c, d and e had significant (*P*<0.05) differences between different salt dust treatment groups, while samples with matching lowercase letters had no significant differences (*P*>0.05). The same below.

### Distribution of the salt anions on the cotton leaves

**Fig 6:**
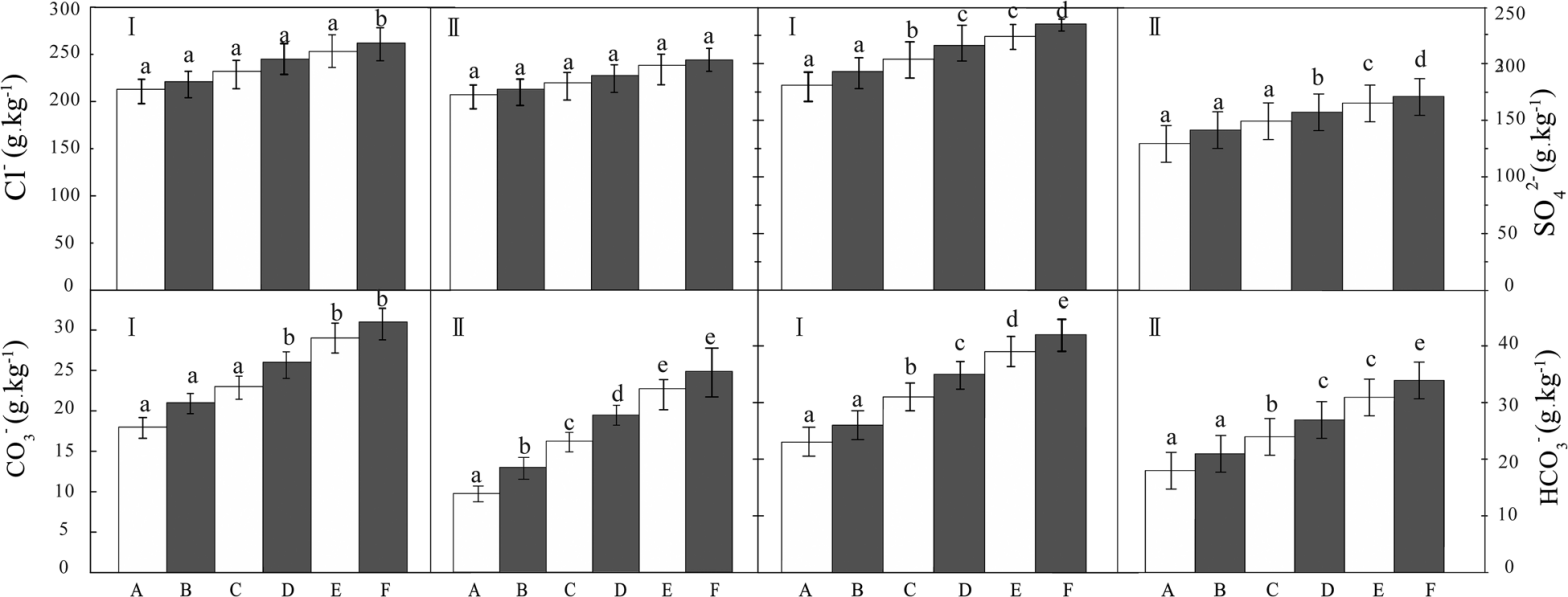
Distribution of the salt anions on the cotton leaves.

From the analysis (Fig 6), the salt anions on the surface of and inside the cotton leaves of control and five salt dust treatment groups were ordered as Cl^-^>SO_4_^2-^>HCO_3_^-^>CO_3_^-^. In the five salt dust treatment groups, the order of the total salt content on and in the cotton leaves was 500 g.m^-2^>400 g.m^-2^>300 g.m^-2^>200 g.m^-2^>100 g.m^-2^.

ANOVA showed that the salt dust deposition had significant influence on the salt anionic contents of the surface cotton leaves (*P*<0.05), and the differences among these groups were significant. Then the further LSD test showed that compared to the control, the Cl^-^ on the surface of the cotton leaves of the treatment groups showed an increasing trend. Specifically, there were significant differences between groups (A, B, C, D, and E) and F (*P*<0.05), but there were no significant differences between groups A, B, C, D, and E. Inside the cotton leaves, there was an increase in Cl^-^ in the treatment groups compared to the control, but there were no significant differences between groups A, B, C, D, E, and F (Fig 6).

The salt anion SO_4_^2-^ content on the surface of the cotton leaves increased in the five treatment groups compared to the control with significant differences between groups (A, B and C), and (D, E), and F (*P*<0.05), but there have no significant differences between groups (A, B and C) and (D, E), respectively. Inside the cotton leaves, the SO_4_^2-^ content increased in the five treatment groups compared to the control, but there were no significant differences between groups A, B, and C.

The LSD test showed an increase in CO_3_^-^ on the surface of the cotton leaves of the five treatment groups compared to the control with significant differences between groups (A, B, C) and (D, E, F) (*P*<0.05), but there were no significant differences between groups (A, B, C) and (D, E, F), respectively. Inside the cotton leaves, the CO_3_^-^ increased in the five treatment groups compared to the control, but there were no significant differences between groups of A and B and C, and D and (E, F) (*P*>0.05).

An increase was observed in content of HCO_3_^-^ on the cotton leaf surface in the salt dust treatment groups compared to the control. There were significant differences between groups (A,B) and C, and D, and E and F (*P*<0.05). Inside the cotton leaves, the CO_3_^-^ showed an increasing trend in the five treatment groups compared to the control, but no significant differences were observed between groups (A,B) and (E,F).

### Changes in the photosynthesis of the cotton leaves

#### *P*_n_ and *PE* changes in the cotton leaves

A bimodal curve of the net photosynthetic rate (*P*_*n*_) was observed when testing the photosynthesis of the cotton leaves, where a "lunch break" phenomenon occurred at 16:00. The curves of the net photosynthetic rate (*P*_*n*_) and the light use efficiency (*PE*) decreased as the salt dust sediment increased from 100g.m^-2^ to 500g.m^-2^.

**Fig 7:**
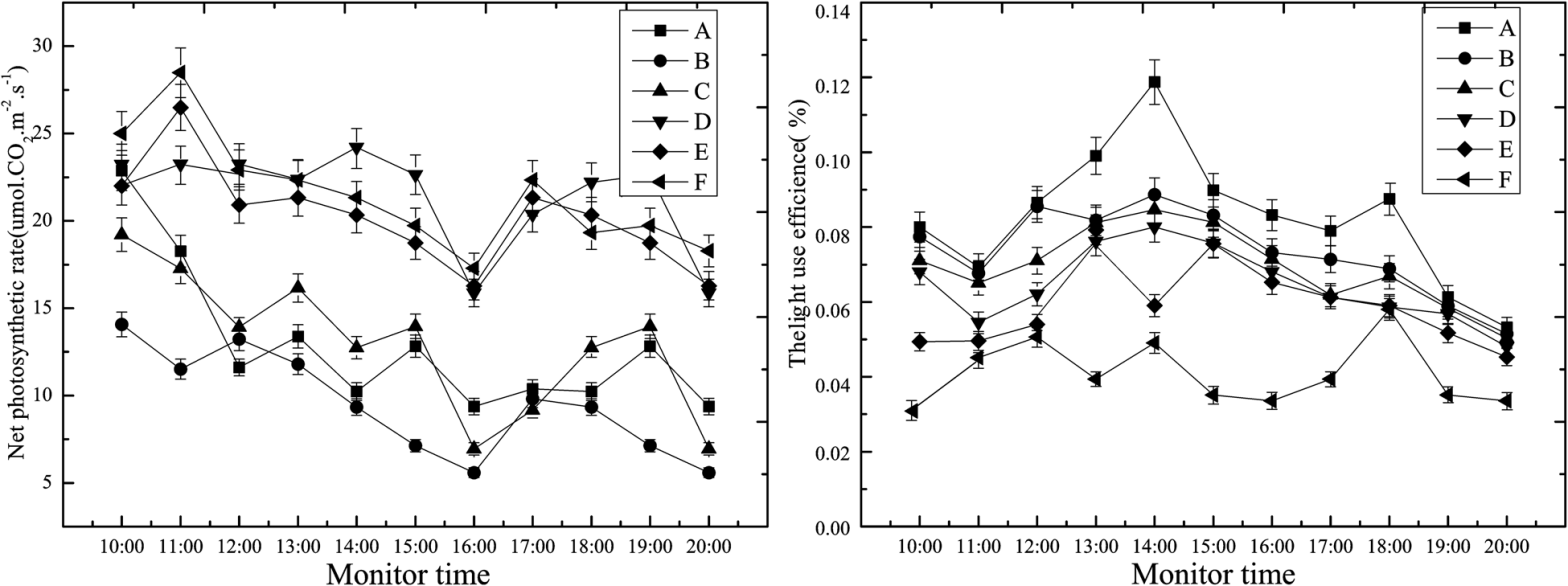
Effect of different salt dust treatments on the net rate of photosynthesis (*P*_*n*_) and efficiency of light use (*PE*) by the cotton leaves.

Compared to the control area, the *P*_*n*_ and *PE* curves were lower and became smaller in the five salt dust sedimentation areas with significant differences in the average value per day (Fig 7; Table 1). This suggests that higher amounts of salt dust had more influence on *P*_*n*_ and *PE* of the cotton leaves [31,32]. The *P*_n_ and *PE* values of the cotton leaves in the salt dust treatment groups all showed a decreasing trend compared to the control with significant differences between groups A and (B, C), and D, and E, and F (*P*<0.05), but no significant differences were observed between groups B and C.

**Table 1:** Average gas exchange indexes of the cotton blades of the control and five salt dust treatment groups (Mean ± SE) (n = 6).

Groups	*P*_n_	*PE*	*T*_r_	*WUE*	*G*_s_	*L*_s_	*C*_*i*_	*T*_*l*_
A:CK	22.53±3.54a	0.056±0.082a	8.45±1.16a	5.78±0.022a	0.591±0.087a	0.692±0.161a	248.7±3.54a	34.43±0.082a
B:100g.m^-2^	20.43±0.45b	0.048±0.002b	8.25±0.29b	5.35±0.002b	0.554±0.002b	0.644±0.002b	218.43±4.45b	32.14±0.002b
C:200g.m^-2^	19.21±0.84b	0.045±0.001b	8.18±0.15b	5.21±0.002b	0.501±0.003b	0.5953±0.003b	209.51±4.84b	31.72±0.001b
D:300g.m^-2^	18.45±0.56c	0.041±0.004c	7.94±0.84c	4.86±0.005c	0.452±0.006c	0.522±0.048c	181.75±7.56c	29.41±0.004c
E:400g.m^-2^	17.13±0.21d	0.038±0.003d	7.16±0.96d	4.01±0.004d	0.431±0.007c	0.499±0.042c	172.13±4.21c	28.21±0.003c
F:500g.m^-2^	16.21±0.64e	0.034±0.003e	6.82±0.86d	3.71±0.004d	0.356±0.007d	0.441±0.054d	136.21±6.64c	27.32±0.003c

Samples within one index with different lowercase letters a, b, c, d and e had significant differences between different salt dust treatment groups(*P*<0.05), while samples with matching lowercase letters had no significant differences.

### Transpiration rate *(T*_*r*_ *)* and water use efficiency *(WUE)* changes in the cotton leaves

**Fig 8:**
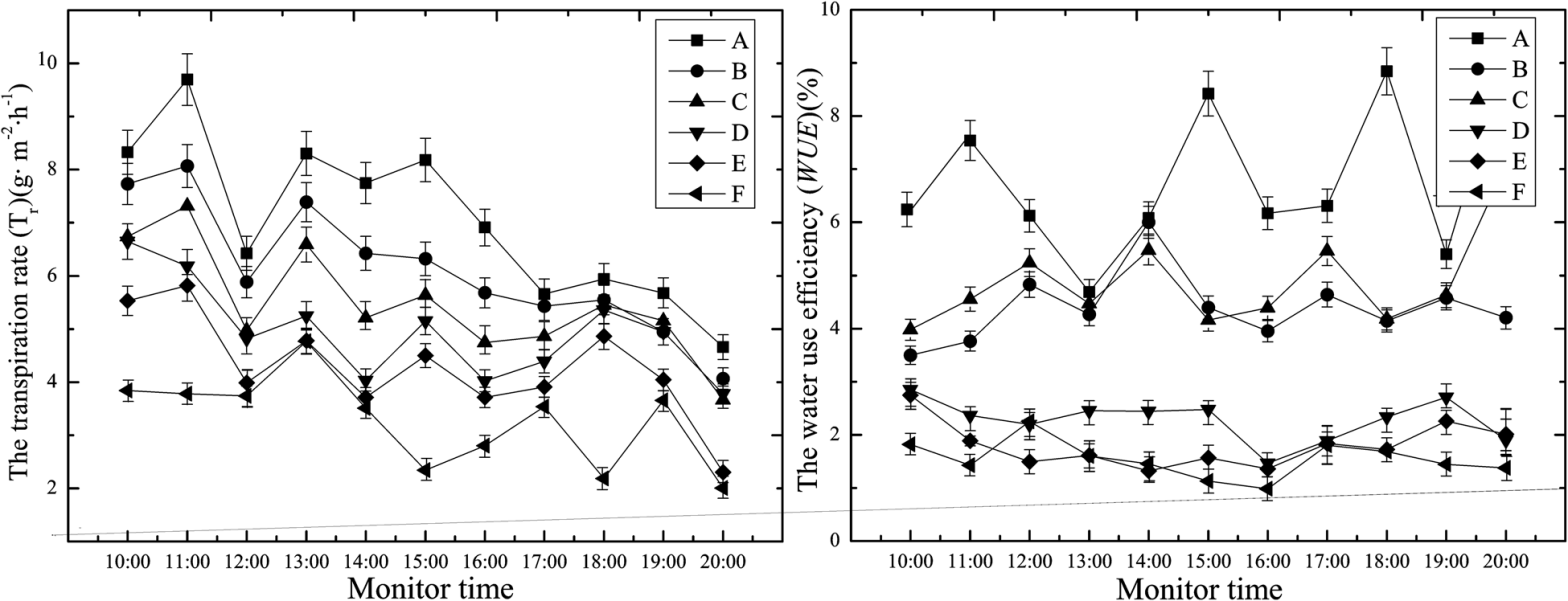
Effect of different salt dust treatments on the rate of transpiration (*T*_*r*_) of and efficiency of water use (*WUE*) by the cotton leaves.

As shown in Fig 8, ANOVA showed that the salt dust deposition had significant influence on *T*_r_ and *WUE* values of the surface cotton leaves (*P*<0.05), and the differences among these groups are significant, then the LSD test showed that (Table 1), *T*_r_ and *WUE* of the cotton leaves decreased in the five salt dust treated groups compared to the control with significant differences between the groups of A, and (B and C), and (D), (E and F) (*P*<0.05). However, there were no significant difference in *T*_r_ and *WUE* of the cotton leaves between salt dust treated groups (B and C), and (E and F), respectively.

### Changes in the *G*_*s*_ and *L*_*s*_ of the cotton canopy

**Fig 9:**
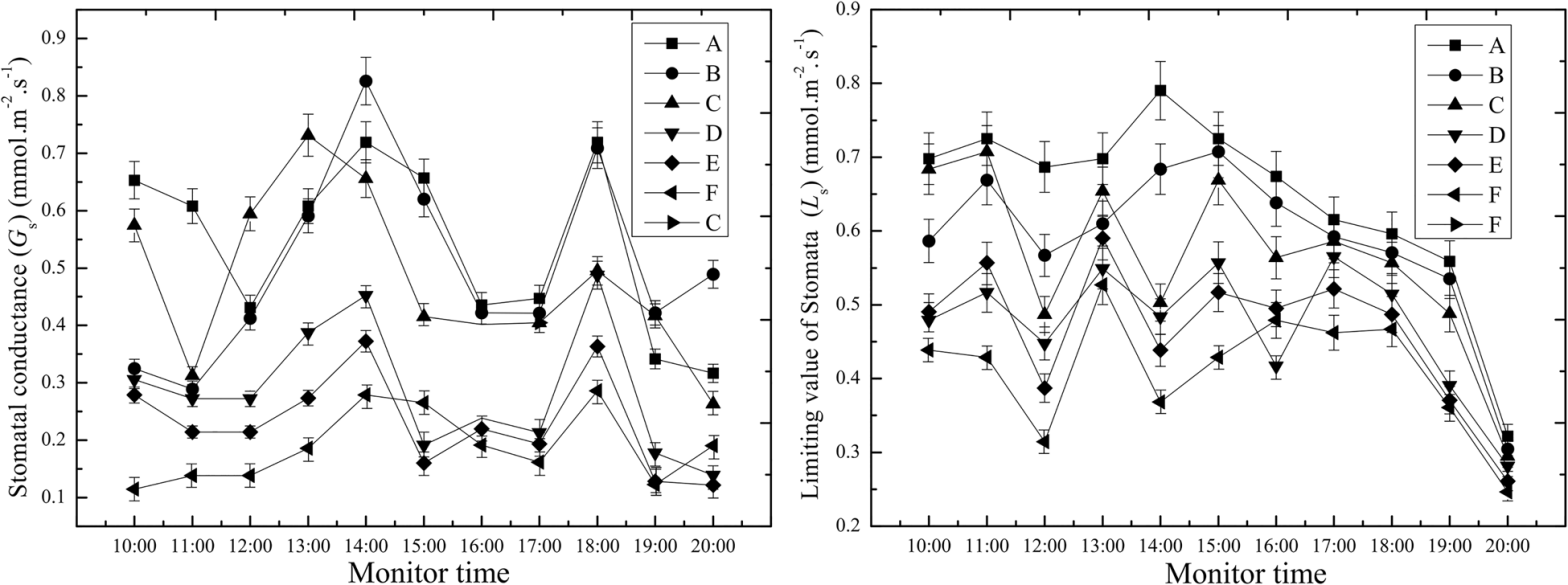
Effect of different salt dust treatments on the stomatal conductance (*G*_*s*_) and the limiting value of the stomata (*L*_*s*_) of the cotton leaves.

As shown in Fig 9, the *G*_s_ and *L*_s_ of the cotton leaves in all the groups displayed an “M” phenomenon. This analysis showed that compared to the control, the *G*_s_ and *L*_s_ of the five salt dust treatment groups decreased from 100 g.m^-2^ to 500 g.m^-2^. The LSD test showed that there were significant differences in the daily mean *G*_s_ and *L*_s_ values between groups A and (B and C), and (D and E) and (F) (*P*<0.05), but there were no significant differences between the salt dust groups (B and C), and (D and E), respectively.

### Changes in the *C*_*i*_ and *T*_*l*_ of the cotton canopy

**Fig 10:**
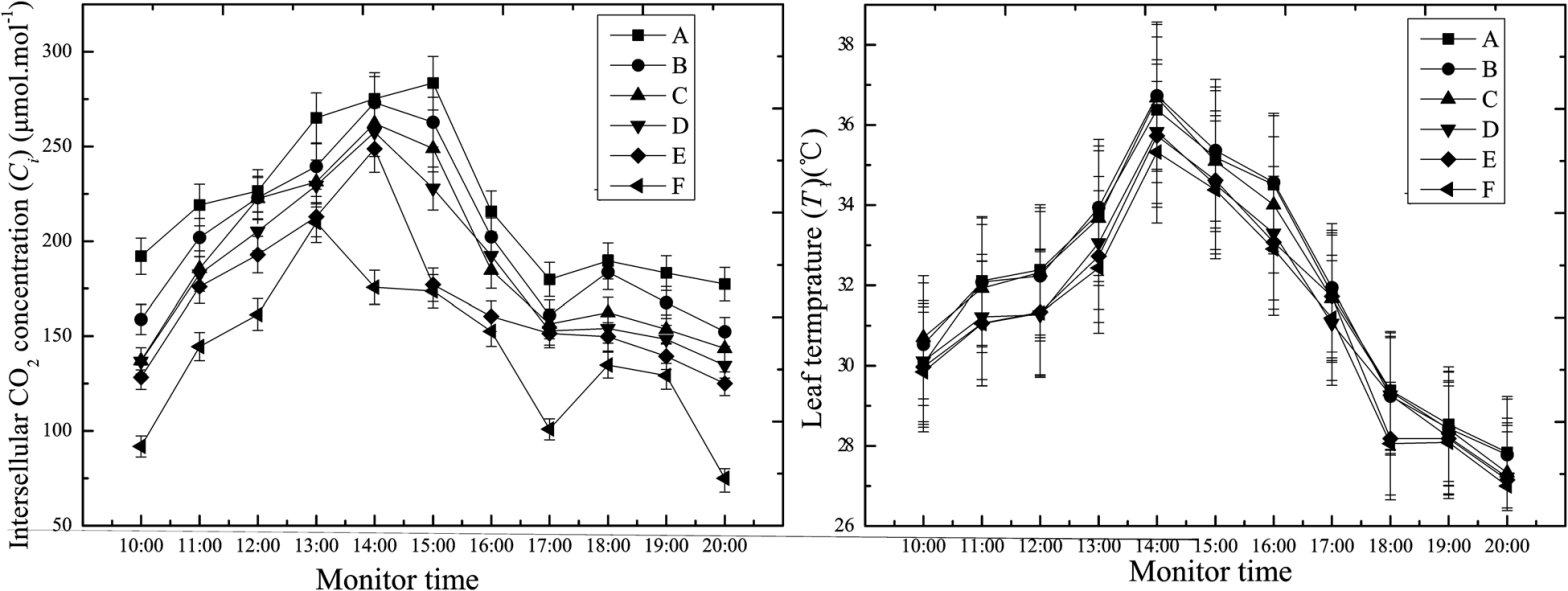
Effect of different salt dust treatments on the intercellular CO_*2*_ concentration (*C*_*i*_) and temperature (*T*_*l*_) of the cotton leaves.

As shown in Fig 10, there was a decrease in the *C*_i_ and *T*_i_ each day in the salt dust treatment groups compared to the control. All the groups displayed a “single peak” phenomonon for the *C*_i_ and *T*_*i*_. The further LSD test showed that there were significant differences between the control and five salt dust sediment groups (*P*<0.05) (Table 1), with significant differences between groups A and (B and C), and (D, E and F)(*P*<0.05), but no significant differences between (B and C), and (D, E and F) groups, respectively.

### Changes in the light response model parameters of the cotton canopy

In order to determine the impact of the salt dust on the cotton canopy, we used the light response curve model [26,33]. First, we calculated the maximum net photosynthetic rate (*A*_max_) and the related parameters and we generated a straight line parallel to the X-axis. Then, we calculated the intersection point of these two straight lines, which corresponds to the light saturation point. The results showed significant differences in the curved angle (*K*), apparent quantum efficiency (*φ*), net photosynthetic rate (A_max_) and respiratory rate (*R*_*ady*_) between the control and five salt dust treatment groups. The *K*, *φ*, *A*_*max*_ and *R*_*ady*_ all decreased in the five salt dust treatment groups going from 100g.m^-2^ to 500 g.m^-2^. ANOVA showed that the salt dust deposition had significant influence on *K*, *φ*, *A*_*max*_ and *R*_*ady*_ values of different groups (Table 2), the LSD test showed that among five salt dust groups and the control group, there were significant differences between A and (B, C) and D, and E and F groups (*P*<0.05), but there was little difference between B and C groups (100 g.m^-2^ and 200 g.m^-2^).

**Table 2:** The light response model parameters of the cotton leaves.

Salt dust sediment groups	Net photosynthetic rate (*A*_max_)	Curved Angle (*K*)	Apparent quantum efficiency (¢)	Respiratory rate (*R*_*day*_)
A:Contrast B:100g.m^-2^	39.6a 36.6b	0.642a 0.581b	0.783a 0.681b	5.94a 5.27b
C:200g.m^-2^	35.9b	0.562b	0.652b	5.02b
D:300g.m^-2^	33.4c	0.474c	0.443c	4.34c
E:400g.m^-2^	31.2d	0.396d	0.376d	3.71d
F:500g.m^-2^	28.2e	0.289e	0.259e	2.98e

Samples within one index with different lowercase letters a, b, c, d and e had significant differences between different salt dust treatment groups, while samples with matching lowercase letters had no significant differences.

### Changes in the chlorophyll, carotenoid, soluble sugar and proline content in the cotton leaves

**Fig 11:**
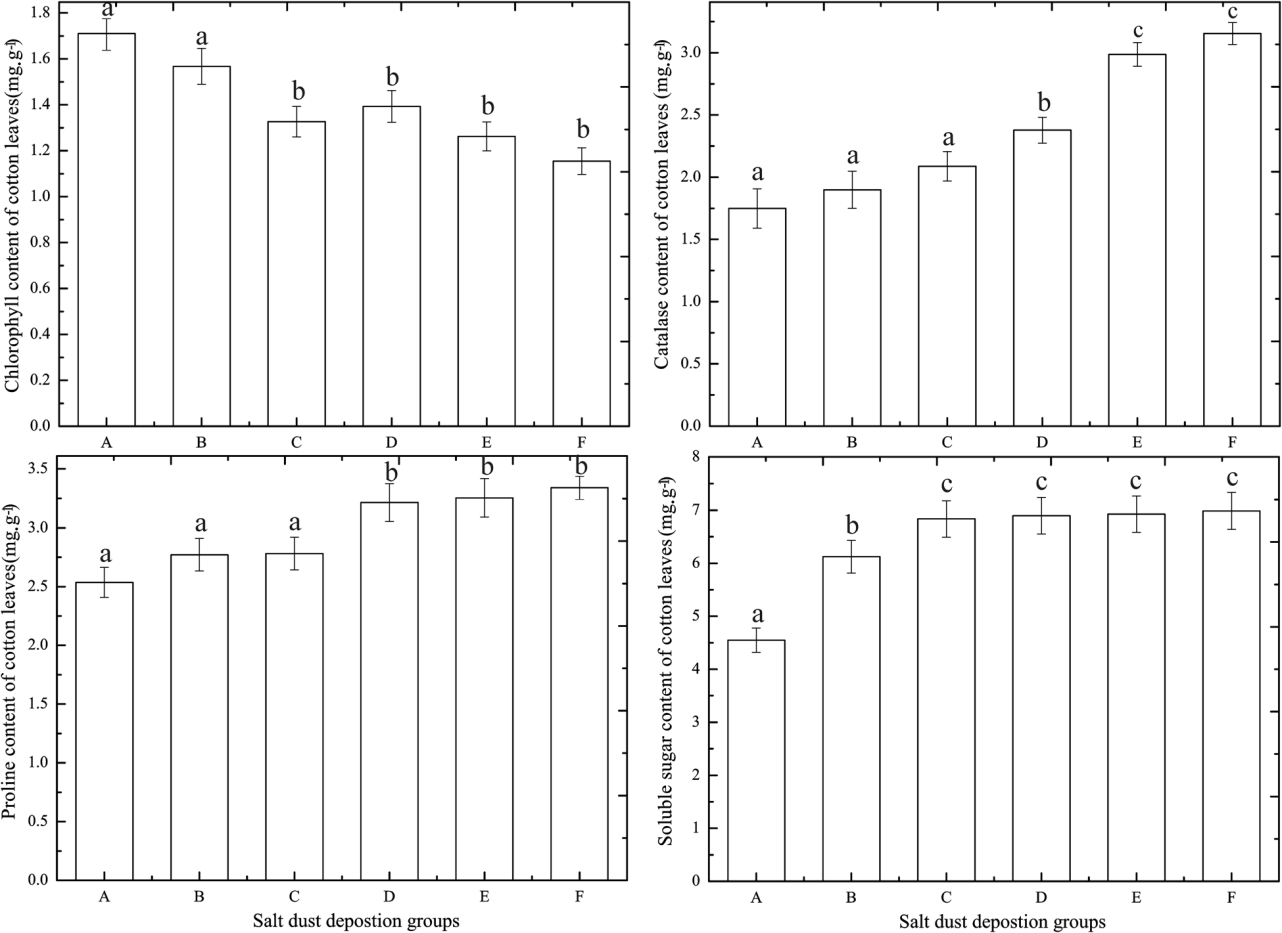
Changes in quantities of chlorophyll, carotenoids, soluble sugars and proline in the cotton leaves.

The amount of chlorophyll in plant leaves can directly influence the photosynthetic intensity to a certain extent. The discretion of the chlorophyll content of the plant leaves is also an important symbol about the leaf function continuous length. By testing the total chlorophyll content in the cotton leaves from different salt dust treatment groups, we found that, compared to the control group, the levels of chorophyll decreased for the five salt dust deposition groups from 100 g.m^-2^ to 500 g.m^-2^. The most significant difference appeared between 100 g.m^-2^ and 200 g.m^-2^ (*P*<0.05). Among the five salt dust groups and the control group, there were significant differences in the mean value of the chlorophyll beween groups (A, B) and (C, D, E and F)(Fig 11), but no significant differences were observed between the salt dust groups (A, B) and (C, D, E, and F), respectively.

Normally, carotenoids are widely distributed and heavily synthesized during higher plant photosynthesis and in photosynthetic tissues, including leaves, flowers, fruit and roots, as well as in microbes, including algae and photosynthetic bacteria. These carotenoids are important photosynthetic auxiliary materials that can supplement the chlorophyll content of the plant leaves, and assist during plant photosynthesis [17,34]. How high or low the carotenoid content in a plant can be an indicator of the strength of the plant leaf photosynthesis. By measuring the carotenoid levels of the cotton leaves from different salt dust treatment groups, we found that with increasing salt dust deposition, the carotenoid content increased as compared to the control. Among five salt dust groups and the control group, there were no significant differences between groups B, C, D, E and F (Fig 11).

Soluble sugars are the primary products of photosynthesis in higher plants, they can form the starch, fat, protein, fiber and other compounds of the plant, and they can maintain the carbon and nitrogen metabolism during the growth period of the plants. They are also part of a class of osmotic regulation substances, which are known to play a significant role in plant growth and development [32].

By testing the soluble sugar in the cotton leaves of salt dust treatment groups and the control, we found that as salt dust deposition increased, the soluble sugar content of the cotton leaves also increased with the most significant differences appearing between the 0 g.m^-2^ and 100 g.m^-2^ groups (*P*<0.05) (Fig 11). Among five salt dust groups and the control group, there were significant differences in the mean value of the the soluble sugar content beween groups A, and B, and (C, D, E and F), but there were no significant differences between groups C, D, E and F), respectively.

Proline is one of the most widely distributed osmotic substances, and, under stress, the plants increase the synthesis and decrease the degradation. This result in an accumulation of a large amount of proline in the plant body that can help adjust the osmotic balance and reduce the harm to the plants caused by osmotic stress by scavenging free radicals and protecting the plant cell structure [34].

From this analysis, it was determined that with increased salt dust deposition, the proline content in the cotton leaves showed increasing trends with the most significant difference appearing between 200 g.m^-2^ and 300 g.m^-2^ (*P*<0.05) (Fig 11). Among five salt dust groups and the control group, there were significant differences in the mean value of the proline content beween groups (A, B and C), and (D, E and F), there were no significant differences between the salt dust groups (A, B and C) and (D, E and F), respectively.

### Changes in the MDA content and the activity of antioxidant enzymes in the cotton leaves

MDA is common membrane of lipid peroxide and can be an indicator of the plant membrane lipid peroxidation, which can change under conditions of stress such as drought, high temperature and salt stress, under which the active oxygen balance is disrupted and plant cells are damaged. Therefore, the MDA content in plant can reflect the degree of the membrane lipid peroxidation of plant cells.

**Fig 12:**
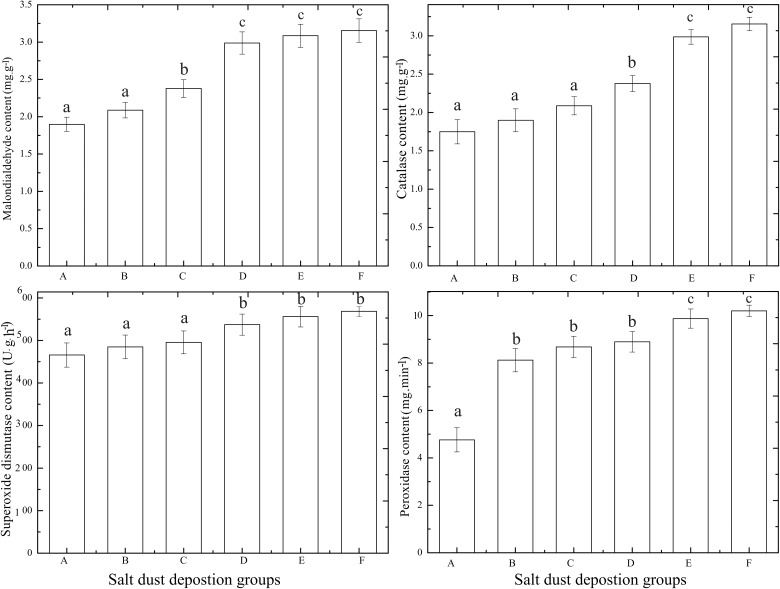
Changes in malondialdehyde (MDA) and antioxidant enzyme activity in the cotton leaves.

The analysis performed in this work determined that when the salt dust deposition increased, the MDA content of the cotton leaves increased with the most significant difference occurring between 200 g.m^-2^ and 300 g.m^-2^ (*P*<0.05) (Fig 12). Among five salt dust groups and the control group, there were significant differences in the mean value of the MDA content beween groups (A, B), and C, and (D, E, and F), while there were no significant differences beween groups (A, B), and (D, E, and F), respectively.

SOD is a protein with physiological activity that can eliminate harmful metabolites. It is also an antioxidant, which can prevent and repair the damaged cells, and remove free radicals from the plant, especially the oxygen free radicals, and oxidize them as peroxide oxygen. Therefore, the SOD activity in the plant tissue can reflect the strength of the plant resistance when in a stressful environment [35].

In this work, it was found that with increased salt dust deposition, the SOD content of the cotton leaves increased with the most significant difference appearing between 200 g.m^-2^ and 300 g.m^-2^ (*P*<0.05). Among five salt dust groups and the control group, there were significant differences in the mean value of the SOD content beween groups (A, B and C) and (D, E and F) (*P*<0.05), but no significant differences were found between groups (A, B and C) and (D, E and F), respectively.

POD is a redox enzyme that uses peroxide as an electron acceptor, and is oxidized by the catalysis of substrates. It can also catalyze peroxide and oxidize amines to remove the toxicity of the amine and peroxide oxygen. It also has a close relationship with respiration and photosynthesis in plants [36].

This analysis found as salt dust deposition increased from 100 g.m^-2^ to 500 g.m^-2^, the POD content in the cotton leaves increased with the most significant difference appearing between 0 g.m^-2^ and 100 g.m^-2^ (*P*<0.05). Among five salt dust groups and the control group, there were significant differences in the mean POD values beween groups A, and (B, C, and D), and (E and F), but there were no significant differences between the groups of (B, C and D) and (E and F), respectively.

CAT resides in the chloroplasts, mitochondria and endoplasmic reticulum of cells, and is especially concentrated in the body of POD. It is an enzyme scavenger that can catalyze peroxide oxygen into oxygen and water, thereby removing peroxide oxygen from the plant body and preventing H_2_O_2_ poisoning of the cells. Therefore, it has antioxidant properties.

This analysis found that as salt dust deposition increased from 100 g.m^-2^ to 500 g.m^-2^, the CAT content in the cotton leaves increased with the most significant difference appearing between 300 g.m^-2^ and 400 g.m^-2^ (*P*<0.05). Among five salt dust groups and the control group, there were significant differences in the mean value of the CAT content beween groups (A, B and C), and (D), and (E, F), there were no significant differences were observed between groups (A, B and C), (E and F), respectively.

## Discussion

By testing the salt ion content, it was determined that the composition and sizes of the particles in the salt dust were similar to the physical and the chemical properties of the natural salt dust as described by Liu et al. [1] and Jilili and Mu [2]. This indicates the salt dust used in these experiments reflects the natural salt dust found on cotton leaves in the region of interest. Using SEM imaging, it was seen the salt dust sedimentation covered an area on the cotton leaves and directly affected photosynthesis, respiration, transpiration and stomatal conductance. Furthermore, the smaller particles in the salt dust could fall into the stomata of the cotton leaves, directly conveying the salt ions and other elements, such as heavy metals, into the plant leaves, and increasing the salt ions inside the cotton leaves. Work by Micklin [37] found that the dust can cover areas on plant leaves, which can cause a significant decrease in photosynthesis by the cotton leaves. McTainsh and Strong [38] determined that tiny dust particles can cover plant leaf stomata specifically and, thereby, affect foliar respiration, photosynthesis and transpiration in the plant leaves. A study by Gao et al. [39] showed that after dust fell into the plant stomata, salt ions within the salt dust entered the plant and joined into the plant nutrient cycle, thus increasing the Na^+^ and Cl^-^ content inside the plant leaves. Previous work revealed that, if the salt ions forming the dust on the surface of the plant leaves reach a threshold value inside the plant leaves, ions are produced that poison the plants and harm plant normal physiological functions, thus influencing plant growth and development.

From the analysis performed in this work, we determined the order of the salt ions inside the cotton leaves of the control and five salt dust treatment groups: Na^+^>Ca^2+^>Mg^2+^>K^+^, while the order of the salt anions on the surface and inside the cotton leaves was Cl^-^>SO_4_^2-^>HCO_3_^-^>CO_3_^-^. It was found that in this work, due to their high permeability, Na^+^ on the surface of the cotton leaves was the large contributor to the salt ion content inside the cotton leaves [18]. This is in contrast to the high amount of K^+^ on the surface of the cotton leaves, yet little K^+^ inside the cotton leaves [31,41]. According to work done by Tuerxun et al. [19], when the soil supply is deficient in potassium, there will be a low concentration of K^+^ in the plant cells. In order to maintain normal cell turgor pressure and functioning of the plant, Na^+^ contained within the plants can partially make up for the lack of K^+^ and relieve the symptoms of potassium deficiency, suggesting an accumulation of Na^+^ inside the leaves. Previous research has shown that K^+^ ions have low permeability, therefore, when salt dust lands on the surface of the cotton leaves, it enters inside the cotton leaf at a lower rate than the Na^+^ ions. This also indicates the K^+^ inside the leaves may come from the soil after being absorbed by the cotton plant roots [40,41].

By comparison, Cl^-^ and SO_4_^2-^ can be easily absorbed by the cotton leaves [17,38,42–44]. In this study, we also found a higher content of Cl^-^ inside the cotton leaves that came from salt dust settling on the surface of the cotton leaves. Additionally, Cl^-^ may come from the soil when absorbed by the cotton plant roots and transferred to the leaves. This may explain why in this study some of the cotton leaves turned yellow in the 500 g.m^-2^ salt dust deposition group. The high content of Na^+^ and Cl^-^ absorbed by the roots from the soil and salt dust on the surface of the cotton leaves accumulated and turned the plant leaves yellow, which is consistent with previous research [17,38,45,46].

In this work, we found little difference between the HCO_3_^-^ and CO_3_^-^ on the surface and inside the cotton leaves, and the overall amount present was very low on both the surface and inside the leaves, indicating HCO_3_^-^ and CO_3_^-^ on the surface of the cotton leaves contributed little to the inside of the cotton leaves. Previous studies have found that HCO_3_^-^ and CO_3_^-^ have low permeability and, therefore, do not get inside the plants as easily as Na^+^ and Cl^-^ [19,47].

Plant leaf photosynthesis is very sensitive to environmental changes [48–50] and can be influenced by many environmental factors, such as light intensity, temperature, moisture and salt stress [36,51–53]. Salt dust deposition poses a challenge to the plant. Previous work has shown that when a certain amount of substance landed on the plant surface, plant photosynthesis was affected [5,31,32]. In this work, we determined that salt dust can have a significant impact on the physiological characteristics of cotton, and, compared to the control, there were significant changes in the photosynthetic characteristics indexes of *P*_n_, *PE*, *C*_i_ *T*_i_, *G*_s_, *L*_s_, *T*_r_ and *WUE* of the cotton leaves, as well as in *K*, *φ*, *A*_max_ and *R*_*ady*_ in the cotton leaves from the salt dust treated groups. Many studies have found that an increase in salt ion content in the sand and dust that landed on the surface of the plant leaves causes a decreae in most of the physiological indexes, such as *P*_n_, *G*_s_, *L*_s_, *C*_i_, *PE*, *K*, *φ* and *A*_max_ [20,26,31,32,54]. In this work, compared with the control, there was an increased effect on the photosynthetic traits of the cotton leaves as the salt dust treatment increased, which was even obvious between the five salt dust groups. Observations made during the experiments found that there were yellow cottons leaves in the five salt dust treatment groups, indicating the salt dust not only influenced the cotton’s photosynthetic abilities, but also the internal physical properties of the cotton leaves [34,55].

The blades are the source organs of the plants, and undertake the substance production that supplies the whole plant. They also have a significant influence on the growth and development of the crops. Previous research has shown that when dust landed on the plant leaves it can form an adhesive layer that can seal the stoma and hinder the respiration, photosynthesis and transpiration of the plant. In this work, we found that in the salt dust treated groups, the respiration, photosynthesis and transpiration of the cotton leaves decreased. In comparison to the control group, the stomatal conductance and limitation of the cotton leaves of the salt dust treated groups all decreased, indicating a decrease in the respiration, photosynthesis and transpiration of the cotton leaves from this. Chen et al. [49] measured the physiological and ecological responses of 22 plants to the dust and found that in some plants, the rate of plant transpiration dropped significantly, while it significantly rose in others due to the adaptation of the plant leaves to the dust in a short period of time. In this study, we found that when the cotton leaves suffered from salt dust deposition, the respiration, photosynthesis and transpiration of the cotton leaves all decreased, which likely reflects the adaptation of the cotton leaves to the salt dust stress in a short period of time. Because landing of salt dust on the surface of the cotton blade can block the absorption of CO_2_ by cotton stomata, there is a reduction in the stomatal conductivity that will eventually influence respiration, photosynthesis and transpiration of the cotton leaves.

The total chlorophyll and carotenoid content of the cotton leaves plays an important role in the photosynthesis of the plants and will influence the normal growth and productivity. Studies performed both at home and abroad have shown that the total chlorophyll and carotenoid content decreased when the plant suffered from stresses such as drought, salinity, and low and high temperatures [40,41,56,57]. Work by Pandey and Sinha [58] found that the total chlorophyll content significantly decreased when the maize leaves encountered coal smoke pollution. This also occurs in wheat leaves when they undergo cement dust deposition. Work by Migahid et al. [59] found that cement dust deposition can result in a significant reduction in the total chlorophyll and carotenoid content of soybean leaves. In this work we found that salt dust deposition on cotton leaves led to a decrease in the total chlorophyll and carotenoid content of the cotton leaves. This is consistent with the above described research, indicating that salt dust deposition significantly influences the photosynthesis of cotton leaves, which will eventually influence cotton production.

Carbohydrates are important components of plants. Soluble sugar is the primary product of photosynthesis in higher plants and it can form the starch, fat, protein, fiber and other compounds found in the plant. It can also maintain the carbon and nitrogen metabolism during the growth period of plant. Furthermore, it is in the class of cell osmotic regulation substances, and, therefore, plays a significant role in plant growth and development. Previous research has shown that when plants suffer from salt stress, arid conditions or freezing, the soluble sugar content of the plants will increase [31,32]. In this work, we found that compared to the control group, the soluble sugar content of the cotton leaves in the salt dust treated groups increased as the salt dust deposition led to the increase in sugar content was more significantly. In this work, Na^+^, Cl^-^, and SO_4_^2-^ within the salt dust landing on the surface of the cotton leaves strongly contributed to the salt ion content inside the cotton leaves. This resulted in stress from the salt ions on the cotton leaves and led to water loss in the cotton cells through an increase in the soluble sugar content to adjust the osmotic potential in an effort to maintain normal physiological functioning of the cells.

Currently, research on the influence of stresses on the cytoplasm membrane structure and function of the plant often focus on increasing MDA content, which is directly related to the degree of injury to the plant cells. In this work, in comparison to the groups that weren’t exposed to salt dust, the leaves exposed to salt dust had an increased MDA content, suggesting the salt dust deposition significantly damaged the cells of the cotton leaves. This led to the production of harmful substances, such as the reactive oxygen species (O_2_^-^, OH^-^, H_2_O, OH^-^, etc.) in the blade cells, which can cause membrane lipid peroxidation, thus destroying the membrane structure of the plant cells. A number of studies have shown that in cases of stress, such as that from salt, drought or high temperature, the MDA content of the plant cells will increase through enhancement of antioxidant enzyme activity to remove the harmful material of reactive oxygen species to reduce the negative influence on plant cell functioning [19,45]. In this work, we found that compared to the control group, the antioxidant enzymes of SOD, POD and CAT were increased in the salt dust treatment groups. This shows that the salt dust deposition was a stress just like drought, high and low temperatures, and can damage the plant cells and raise the MDA content by enhancing the activity of antioxidant enzymes to remove reactive oxygen species. This is consistent with published work.

This work found that when salt dust landed on the surface of cotton leaves, it impacted the photosynthesis, respiration and transpiration of the cotton leaves, which then affected physiological and biochemical processes, such as nutrient absorption, and chlorophyll and carotenoid content. This can also destroy the structure of the cotton leaves cells, and increase the MDA content of the cotton leaves by increasing osmotic adjustment substances, such as proline and soluble sugar, to reduce cytosolic extravasation. There is also an increase in the activity of antioxidant enzymes, such as the SOD, POD and CAT, to remove reactive oxygen and MDA in the cotton leaves to maintain normal functioning of the cotton leaves.

Over the past 50 years with the rapid land-use changes in the Ebinur Basin, man has significantly affected and modified the natural landscape of this area. These changes increased as the population density increased and agriculture intensified [1,2]. The conversion of land into that used for agriculture, and other driving forces of land-use changes are the leading factors behind the excessive use of water resources in this area, which resulted in a significant reduction in the surface water resources that flow into the Ebinur [1,15]. Overall, in the water area of the Ebinur to the remaining 500 square kilometers that resulted in a large bared area of dry lake bottom. This dry lake bottom has become a huge salt dust storm source in Northwest China and Central Asia [1,4,22,60]. These areas face the serious threat of the salt dust storms. In order to protect the environment and reduce the effect of the salt dust storms on agriculture and economy, we should pay increased attention to these phenomena and take efficient protective measures in this area.

## Conclusion

By studying the influence of salt dust in Ebinur Basin on the salt ion content of cotton leaves on photosynthesis, leaf blade surface morphology, and biochemical processes, we reached the following conclusions:

1. The salt ion content, composition and particle size of the salt dust used in these experiments were similar to those of natural salt dust, suggesting the salt dust on the cotton leaves was from salt dust storms in this region.
2. The primary salt cations on the surface and inside the cotton leaves were Na^+^, Ca^2+^, Cl^-^ and SO_4_^2-^, while the CO_3_^-^ and HCO_3_^-^ contents were present only at low levels. Overall, the salt cation and anion content on the surface and inside the cotton leaves can be ordered as Na^+^>Ca^2+^>Mg^2+^>K^+^ and Cl^-^>SO_4_^2-^>HCO_3_^-^>CO_3_^-^, respectively. There were significant differences in the Na^+^, Ca^2+^, Mg^2+^, K^+^, Cl^-^, SO_4_^2-^, HCO_3_^-^ and CO_3_^-^ found on the surface and inside the cotton leaves of the salt dust treated groups compared to the control, but the differences varied between salt dust groups. Both Na^+^ and Cl^-^ found on the surface of the leaves contributed strongly to the amount inside the cotton leaves. Overall, the five salt dust treatment groups are ordered according to total amount of salt ions on the surface and inside the cotton leaves as: 500 g.m^-2^>400 g.m^-2^>300 g.m^-2^>200 g.m^-2^>100 g.m^-2^.
3. The salt dust that lands on the surface of cotton leaves can significantly influence the photosynthetic traits of *P*_n_, *PE*, *C*_i_ *T*_i_, *G*_s_, *T*_r_, *WUE*, *L*_s_, *φ*, *A*_max_, *k*, and *R*_*ady*_ of the cotton leaves, as the differences between the control and the groups treated with salt dust groups were significant. However, differences between the five salt dust groups varied.
4. Salt dust can significantly hinder the physiological functions of cotton leaves, such as causing a decrease in leaf chlorophyll and carotenoid content, and increasing cytoplasmic membrane permeability and the MDA content. The MDA content is increased by increasing the soluble sugar and proline content to adjust for the loss of the cytosol in the cells, the activity of antioxidant enzymes to eliminate harmful molecules, such as intracellular reactive oxygen species, in an effort to reduce the damage caused by salt dust stress and maintain normal physiological functions. As a whole, salt dust deposition is a hardship for the cotton crops, which is consistent with previously published work concerning the effect of salt dust on other plants.

This study revealed that salt dust can significantly influence the physiological and biochemical processes of cotton leaves, which will eventually cause plant damage and reduce cotton production and, therefore, lead to agricultural economic loss. Therefore, much attention should be paid to the salt dust storms in the Ebinur Basin and efficient measures should be undertaken to protect the environment.

### Measures

In order to reduce the negative effects of salt dust storms in the Ebinur Basin area, we suggest the following measures:

1. Isolation technology should be used to limit the perennial water deposition and dry lake area with the goals of returning farmland to forests, grassland and lake to stabilize and increase the water area of the Ebinur, as well as curb the source of the sand in this area.
2. Biological control techniques should be used to cultivate salt tolerant shrubs and trees, such as *Saxoul*, *Tamarix*, and *Populus euphratica*, in order to decrease the uptake of the salt dust from the dry salt bottom of the lake and the threat of wind erosion in this basin.
3. Strict water management systems should be implemented to reduce the irresponsible use and waste of water resources in the river basin. Specifically, the use of water resources from the Bortala and Jing rivers, which are the main rivers feeding into Ebinur, should be reduced. The exploitation of groundwater in this area should be reduced and precipitation should be artificially enhanced to maintain the water resources in this area and water levels of the lake.
4. New agricultural techniques, such as plastic membrane mulching and drip irrigation, should be used, especially in cotton fields, to improve the use of the limited water resources, and to raise the price lever of the water resources used by industry. Furthermore, industrial projects, such as coal and silicon chemical enterprises, should be restricted as they can be harmful to the natural environment.
5. The Ebinur Basin should be established as a nature reserve in order to strength the soil and water conservation and afforestation, strengthen management of the Ebinur Basin, prohibit deforestation, rotten dig herbs and exploitation of the grassland, and ensure the normal growth and natural regeneration of the natural vegetation, predominantly *Sacsaoul* forest. These measures should reduce the negative influence of the sand from the dried bottom of the Ebinur Basin and, thus, protect the natural environment.
6. Measures should be taken to recover the crop output of crops after damage from the salt dust in the area, such as enhancing the fertilizer with N, P, and K to improve the absorption of the nutrients from the soil and increasing the amount of irrigation, to promote crop productivity and reduce damage to the economy.
